# Supplementary material for: VegVault dataset: linking global paleo-, and neo-vegetation data with functional traits and abiotic drivers
Source: Sci Data. 2025 Dec 5;12:1923. doi: 10.1038/s41597-025-06176-1 (PMC12680632; doi:10.1038/s41597-025-06176-1)
Supplement: Supplementary file 4 — Supplementary File [file 41597_2025_6176_MOESM4_ESM.pdf]

VegVault dataset: linking global paleo-, and neo-vegetation data with functional traits and abiotic drivers

Authors

Ondřej Mottl[1,2], Franka Gaiser[3], Irena Šímová[1,4], Suzette G. A. Flantua[5,6]

Affiliations

1. Center for Theoretical Study, Charles University, Jilská 1, CZ-11000 Prague, Czech Republic
2. Department of Botany, Faculty of Science, Charles University, Benátská 2, CZ-12801 Prague, Czech Republic
3. Sport Ecology, Bayreuth Center of Ecology and Environmental Research (BayCEER) & Bayreuth Center of Sport Science (BaySpo), University of Bayreuth, 95447 Bayreuth, Germany
4. Department of Ecology, Faculty of Science, Charles University, Viničná 7, CZ-12800 Prague, Czech Republic
5. Department of Biological Sciences, University of Bergen, PO Box 7803, N-5020 Bergen, Norway
6. Bjerknes Centre for Climate Research, Bergen, Norway

corresponding author(s): Ondřej Mottl (ondrej.mottl@gmail.com)

Supplementary Tables

Table S1: A list of selected depositional environments used during the processing of fossil pollen data.

Table S2: A list of selected ecological groups used during the processing of fossil pollen data.

Table S3: A list of valid control types used during the age-depth modelling process during the processing of fossil pollen data.
